# Dietary Compounds as Epigenetic Modulating Agents in Cancer

**DOI:** 10.3389/fgene.2019.00079

**Published:** 2019-03-01

**Authors:** Ángeles Carlos-Reyes, José Sullivan López-González, Manuel Meneses-Flores, Dolores Gallardo-Rincón, Erika Ruíz-García, Laurence A. Marchat, Horacio Astudillo-de la Vega, Olga N. Hernández de la Cruz, César López-Camarillo

**Affiliations:** ^1^Laboratorio de Cáncer de Pulmón, Instituto Nacional de Enfermedades Respiratorias “Ismael Cosio Villegas”, Mexico City, Mexico; ^2^Laboratorio de Medicina Traslacional, Instituto Nacional de Cancerología, Mexico City, Mexico; ^3^Programa en Biomedicina Molecular y Red de Biotecnología, Instituto Politécnico Nacional, Mexico City, Mexico; ^4^Laboratorio de Investigación Traslacional en Cáncer y Terapia Celular, Hospital de Oncología, Centro Médico Nacional Siglo XXI, Instituto Mexicano del Seguro Social, Mexico City, Mexico; ^5^Posgrado en Ciencias Genómicas, Universidad Autónoma de la Ciudad de México, Mexico City, Mexico

**Keywords:** phytochemicals, cancer therapy, histones modifications, epigenetic machinery, DNA methylation

## Abstract

Epigenetic mechanisms control gene expression during normal development and their aberrant regulation may lead to human diseases including cancer. Natural phytochemicals can largely modulate mammalian epigenome through regulation of mechanisms and proteins responsible for chromatin remodeling. Phytochemicals are mainly contained in fruits, seeds, and vegetables as well as in foods supplements. These compounds act as powerful cellular antioxidants and anti-carcinogens agents. Several dietary compounds such as catechins, curcumin, genistein, quercetin and resveratrol, among others, exhibit potent anti-tumor activities through the reversion of epigenetic alterations associated to oncogenes activation and inactivation of tumor suppressor genes. In this review, we summarized the actual knowledge about the role of dietary phytochemicals in the restoration of aberrant epigenetic alterations found in cancer cells with a particular focus on DNA methylation and histone modifications. Furthermore, we discussed the mechanisms by which these natural compounds modulate gene expression at epigenetic level and described their molecular targets in diverse types of cancer. Modulation of epigenetic activities by phytochemicals will allow the discovery of novel biomarkers for cancer prevention, and highlights its potential as an alternative therapeutic approach in cancer.

## Introduction

The knowledge of epigenetic mechanisms regulating gene expression has allowed significant advances in the understanding of cancer biology. Cancer is caused by the accumulation of genetic and epigenetic alterations, which induce alterations in the expression of oncogenes and tumor suppressor genes (Sharma et al., 2010; McCleary-Wheeler et al., 2013; Aggarwal et al., 2015). Changes in the DNA sequence are caused by mutations, amplifications or deletions. Moreover, regulation of gene expression is also modulated by epigenetics modifications of chromatin with no alterations in DNA sequence. The main epigenetic mechanisms studied in mammalian cells are DNA methylation and histones modifications which induce remodeling of chromatin resulting in changes of cellular phenotypes (Shukla et al., 2014). In cancer cells, diverse epigenetic alterations of cancer-related genes occur in the early stages of tumor development. Remarkably, these epigenetic modifications of chromatin are inherited and reversible, so they represent promising targets for the development of novel drugs targeting the epigenome which may contribute to amelioration of conventional therapies in cancer (Esteller, 2008; Shukla et al., 2014; Kagohara et al., 2017; Zadi Heydarabad et al., 2018). Improvement in patient’s survival is the result of the use new therapeutic drugs and personalized options. Some of these treatments have been combined with alternative therapies represented by natural phytochemical compounds. It has been reported that a diet rich in vegetables and fruits can significantly reduce the risk of cancer development, due to the action of phytochemicals which may regulate the expression of oncogenes and tumor suppressor genes. Remarkably, phytochemicals may act through epigenetic mechanisms such as modulation of DNA methyltransferases (DNMTs) and histone deacetylases (HDACs) activities (Yang and Zheng, 2014; Zhou et al., 2016). In general, cancer treatments involve the use of chemo-radio therapeutic agents, kinase inhibitors, personalized antibodies as well as compounds that stimulate the immune system. In particular, HDAC inhibitors and demethylating drugs modified gene expressions by reversing the aberrant epigenetic alterations acquired during tumorigenesis (Luczak and Jagodziński, 2006). In this context, phytochemicals may represent an alternative therapeutic option for cancer treatment. In this review, we first provided an overview of the most frequent epigenetic alterations in human cancers, then we described the most studied dietary phytochemicals and their potential use in the reversion of cancer hallmarks through epigenetic mechanisms, and finally we discussed their potential use as an alternative strategy for cancer therapy.

## Epigenetic Mechanisms

### DNA Methylation

DNA methylation is a normal mechanism of gene silencing by transcriptional repression. Global DNA methylation is associated with several processes such as genomic imprinting, X-chromosome inactivation, and the repression of repeated elements. Importantly, dysregulation of these cellular events is frequently found during early and late stages of tumorigenesis (Lim and Maher, 2010; Jin et al., 2011). DNA methylation involves the covalent modification of DNA mediated by DNMT, which transfer methyl groups (-CH3) from *S*-adenosyl-L-methionine (SAM) to cytosine in the so-called CpG islands (Verma and Srivastava, 2002). The CpG regions are contained in large DNA sequences found mainly in gene promoters, intergenic regions and repeated elements (Esteller, 2011; Moore et al., 2013). Aberrant DNA methylation patterns play a crucial role in genomic instability, activation of oncogenes and silencing of tumor suppressor genes involved in cell proliferation, cell cycle, DNA repair, stress response and apoptosis (Esteller, 2005; Ehrlich, 2009; Berdasco and Esteller, 2010; Zaidi et al., 2013; Li et al., 2017a). These alterations are common during the development and progression of carcinogenesis (Esteller and Herman, 2002). Several studies have showed the impact of hypomethylation or hypermethylation of DNA sequences associated with the transcriptional regulation of cancer-related genes (Mossman and Scott, 2006). For instance, LINE-1 gene hypomethylation is associated with different clinical-pathological characteristics in lung cancer; early carcinogenesis in breast cancer, and metastasis in colorectal adenocarcinoma (Estécio et al., 2007; Hur et al., 2014; Park et al., 2014; Imperatori et al., 2017). On the other hand, hypomethylation of specific genes like TTF-3, MUC4, and CT45 frequently occur in prostate, pancreatic and ovarian cancers (Zhu et al., 2011; Zhang W. et al., 2015; Haldrup et al., 2017; Nørgaard et al., 2017). The hypermethylation of RASSF1A gene has been identified as a diagnostic marker in lung cancer, whereas it contributes to increased mortality in women with breast cancer. Hypermethylation of RASSF1A also has been related with high risk of ovarian cancer (El-Sherif et al., 2016; Rezk et al., 2018; Yadav et al., 2018). Other studies showed that hypermethylation of PDE3A gene modulated the response to therapy in cisplatin-resistant non-small cell lung cancer (NSCLC) (Tian et al., 2017). In addition, hypermethylation of genes such as NDN activated the WNT signaling pathway contributing to cell proliferation of colorectal cancer cells (Hu et al., 2017). These examples illustrate the impact of alterations in epigenetic mechanisms in the development of diverse types of human cancers.

### Histone Modifications

Histones are alkaline proteins located in the nucleus of eukaryotic cells (Li, 2012). Their function is to package DNA into structural units called nucleosomes. The core component of nucleosome is the histone octamer, which is formed by two copies of each histone H2A, H2B, H3; whereas histone H1 binds to the linker DNA between nucleosomes (Cutter and Hayes, 2015; Biterge, 2016). The histones have flexible tails that protrude from the lateral surface of the nucleosome. These tails are susceptible to suffer different chemical modifications in the lysine (K), serine (S), threonine (T) and arginine (R) amino acids (Smith and Denu, 2009; Lawrence et al., 2016). Histone modifications in specific amino acids residues (termed histone code) modulate the chromatin structure and gene expression (Karlić et al., 2010; Prakash and Fournier, 2017). Histone proteins have structural and functional roles in the transition between the active and inactive chromatin states. When chromatin is highly packed (heterochromatin) the transcription of genes is blocked; whereas when the chromatin is less condensed (euchromatin) the transcription is activated (Mariño-Ramírez et al., 2005; Bégin and Nadeau, 2014; Glant et al., 2014; Biterge, 2016). Histones can be modified covalently in the N- and C-terminal tails by two families of enzymes; the histone acetyltransferases (HATs) that transfer acetyl groups from coenzyme A (CoA), and the histone deacetylases (HDACs) that remove the acetyl groups from specific amino acid residues (Bannister and Kouzarides, 2011). Histone modifications regulate different biological processes such as transcription, chromosome packaging, and DNA damage response and repair. The primary modifications of histones are acetylation, methylation, phosphorylation, ubiquitination, sumoylation, glycosylation and poly ADP ribosylation (Li, 2012; Marmorstein and Zhou, 2014; Zhang T. et al., 2015; Shanmugam et al., 2018). The acetylation and methylation are well studied mechanisms of histone modifications, and they are key regulators of cellular proliferation, differentiation, division and plasticity (Cohen et al., 2011).

In cancer cells, histones modifications at specific amino acids residues are related to transcription activation or repression. In most species, histone H3 is primarily acetylated at lysines 9, 14, 18, 23, and 56, methylated at arginine 2 and lysines 4, 9, 27, 36, and 79, and phosphorylated at ser10, ser28, Thr3, and Thr11. For instance, gene activation has been associated with trimethylation of lysine 4 on the histone H3 (H3K4me3), acetylation of lysine 9 on the histone H3 (H3K9ac) and monomethylation of lysine 20 on the histone H3 (H3K20me). In contrast, the H3K9me3 and H3K27 epigenetic marks induce repression of gene expression (Leszinski et al., 2012; Bag and Bag, 2018). In prostate cancer, H3K9me2 and H3Ac marks distinguish between tumors from non-malignant tissues, and the H3K4me1 modification was established as a biomarker of tumor progression and a predictor of recurrence after radical prostatectomy (Ellinger et al., 2009). H3K4me and H3K9 modifications were regulated by histone demethylase JMJD2B and promoted hormonal responsiveness in breast cancer patients (Shi et al., 2011). In early-stage of colon cancer (TNM stage I and II) low nuclear expression of H3K4me3 and high expression of H3K9me3 and H4K20me3 were associated with good prognosis (Benard et al., 2014).

## Polyphenols Classification and Functions

**FIGURE 1:**
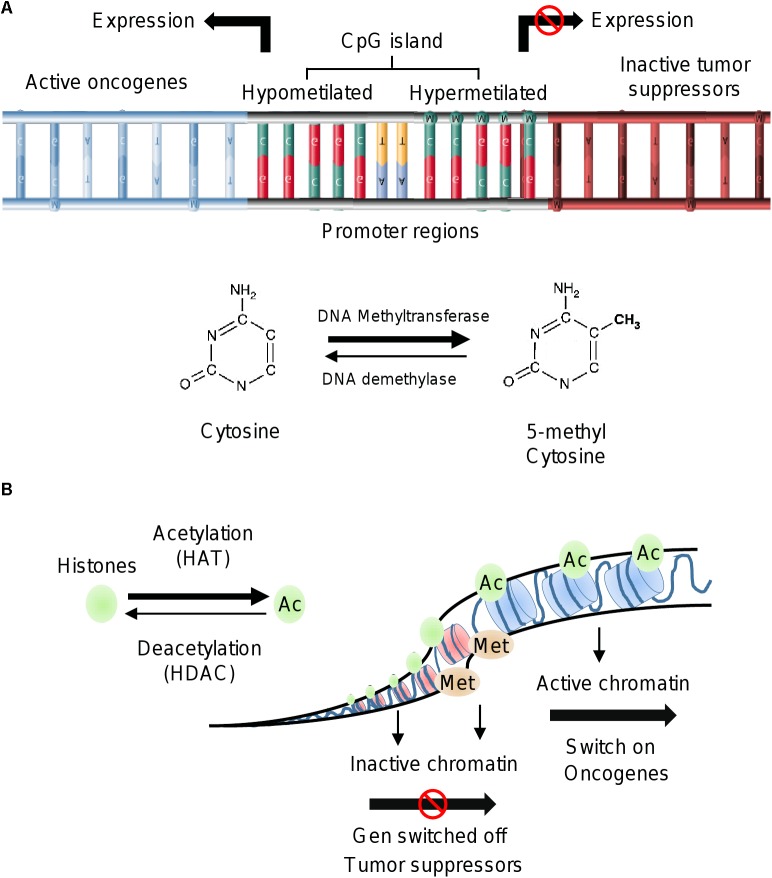
Epigenetic alterations in cancer. **(A)** Methylation of DNA by DNA methyltransferases occurs on CpG islands of promoter gene regions. Hypermethylation of DNA induces repression of gene expression, whereas the hypomethylation status is associated to activation of gene transcription. **(B)** Histone modifications include acetylation by HAT and deacetylation by HDAC. Acetylated status of histones is observed in euchromatin and related to oncogenes activation. Histone methylation can be associated with either transcriptional repression or activation. Trimethylation of histone H3 at lysine 4 (H3K4me3) is an active mark for transcription.

Polyphenols are the most important group of phytochemicals present in plants. They are secondary plant metabolites found in the fruits, vegetables, cereals, and natural beverages. These compounds exhibit biological roles in human cells acting as antioxidants, antimicrobials, detoxification, immune system stimulation, decrease of platelets aggregation, modulation of hormonal metabolism and anti-tumoral agents (Tsao, 2010; Saxena et al., 2013; Jabeena et al., 2014; Ganesan and Xu, 2017). Research in recent years strongly supports a role for polyphenols as important molecules protecting cells from DNA damage (Choudhury et al., 2009; Saxena et al., 2013). Therefore, polyphenols have important benefits in the prevention and treatment of oncologic diseases. Currently, the main polyphenols studied in cancer cells are the phenolic acids, flavonoids, stilbenes, and lignans (Figure 1) (Choudhury et al., 2009; Tsao, 2010; Ganesan and Xu, 2017).

### Phenolic Acids

The hydroxycinnamic acids are the more frequent simple phenolic compounds found in natural sources. The cinnamic acid, *p*-coumaric acid, ferulic acid, caffeic acid, chlorgenic acid, and rosmarinic acid belong to this class. These acids are rarely found as free form but these compounds can found as glycosylated derivatives or esters of quinic acid, shikimic acid, and tartaric acid in processed food that has undergone freezing, sterilization, or fermentation. High concentrations of hydroxybenzoic acid are contained in red fruits, black radish, and onions. Hydroxybenzoic acids form complex structures such as hydrolysable tannins. These tannins also have multiple health benefits including cancer suppression (Tsao, 2010; Hardman, 2014). Combination of caffeic and quinic acid form the chlorogenic acid, which is found in many types of fruit as blueberries, kiwis, plums, cherries, and at high concentrations in coffee. The curcumin acts as antioxidant and anti-inflammatory and has been reported that it has multiple effects in cancer cells (Manach et al., 2004; Hardman, 2014).

### Flavonoids

**FIGURE 2:**
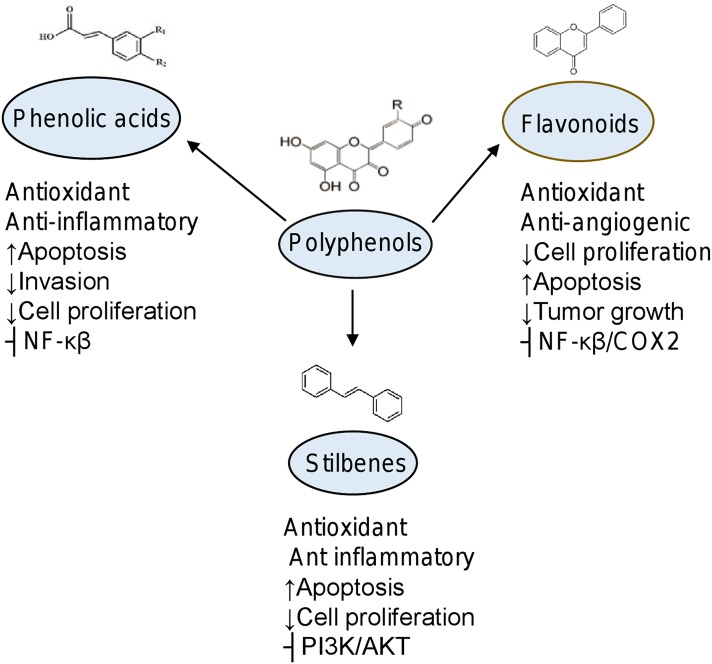
Biological effects of phytochemicals in cancer. Chemical structures of subgroups of polyphenols and organosulfur compounds. Effects in diverse cellular processes are indicated. ⊢, inhibition; ↑, increased; ↓, decreased.

Flavonoids are natural molecules containing variable phenolic structures composed of 15-carbon (C6–C3–C6) skeleton and two benzene rings joined by a linear 3-carbon chain (El Gharras, 2009). Subgroups of flavonoids are: flavonols such as quercetin, kaempferol, and myricetin. They are found in onions, curly, broccoli, and blueberries. The flavanones as eriodictyol, hesperetin, and naringenin are found in grapefruit, oranges, and lemons. The isoflavonoids including daidzein, genistein, and glycitein are presents in leguminous. The flavones as apigenin and luteolin are in cereals. The flavanols as catechin are found in green tea and chocolate, and the anthocyanins including cyanidin, delphinidin, malvidin, pelargonidin, peonidin, and petunidin are present in berries, pears, apples, grapes and peaches (Kawser Hossain et al., 2016). The biological effects of flavonoids have been linked to their anti-obesity, anti-diabetic and anti-oxidative activities. In cancer, these compounds inhibit cell proliferation, induce cytotoxicity, suppression of angiogenesis; and cause cell death by apoptosis (Figure 2) (Chahar et al., 2011; Falcone Ferreyra et al., 2012).

### Stilbenes

Natural stilbenes are non-flavonoid phytochemicals with polyphenolic structure that containing a 1,2-diphenylethylene nucleus. Stilbenes are found in berries, grapes and peanuts. These compounds have a high potential for the prevention and treatment of different diseases. In cancer cells, they showed antioxidant and anti-inflammatory effects, as well as cell death induction. The main stilbene studied to date in cancer is resveratrol (Niesen et al., 2013; Sirerol et al., 2016).

### Sulforaphane

Sulforaphane [1-isothiocyanato-4-(methyl-sulfinyl)] butane is the most widely characterized molecule belong to the isothiocyanate group of organosulfur compounds. Sulforaphane (SFN) is produced by the hydrolysis of glucoraphanin after intake of cruciferous vegetables such as broccoli, cabbages, kale, Brussels sprouts, radish, and mustard. This compound displays anti-inflammatory, antibiotic, and antioxidant activities (Figure 2). In cancer cells, SFN acts as potent chemopreventive and anti-tumoral natural agent (Joozdani et al., 2015; Tortorella et al., 2015).

## Epigenetic Modulation by Polyphenols in Cancer Pathways

**FIGURE 3:**
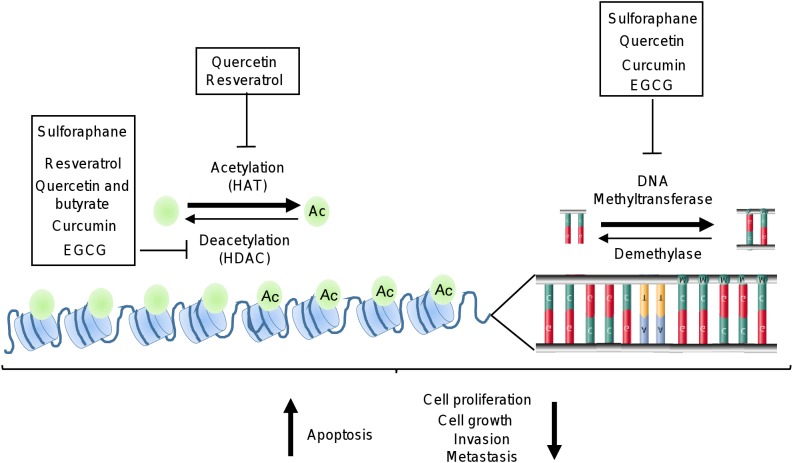
Epigenetic machinery as targets of polyphenols. Illustration depicts how polyphenols may inhibit HDAC, HAT, and DNA methyltransferases activities which are deregulated in cancer cells.

Recent reports indicate that dietary supplements and natural compounds may restore the normal epigenetic marks which are altered during carcinogenesis. The phytochemicals most studied in cancer are epigallocatechin-gallate (EGCG), quercetin, resveratrol, curcumin, and SFN. Several studies have showed that these natural compounds inhibit several cellular processes associated to cancer (Figure 3). In particular, these compounds blocked the development and progression of tumors by targeting key signaling transducers resulting in the restoration of tumor suppressor genes, and inhibition of oncogenes expression (Greenlee, 2012; Guo et al., 2015b; Zhou et al., 2016). These effects are mediated, in part, by the modulation of epigenetic machinery which included the regulation of DNMTs and HDACs activities (Figure 3 and Table 1) (Shukla et al., 2014; Thakur et al., 2014; Li et al., 2015; Deb and Gupta, 2015; Shankar et al., 2016; Sundaram et al., 2017).

**Table 1:** Epigenetic modulated targets by phytochemicals in cancer.

Phytochemical group	Compound	Natural source	Epigenetic modulation	Gene targets	Biological effects	Cancer type	Reference
Phenolic acids	Hydroxybenzoic acid: gallic acid Hydroxycinnamic acid: Caffeic acid, curcumin	Red fruit, onions, black radish, cereals, sage, oregano	DNMT1, DNMT3b, DNMT3a, HDAC1, HDAC4, HDAC7	RASSF1A, RARβ, DLC1, p15INK4b, Nrf2	Chemoprevention, cell growth inhibition, cell cycle arrest, apoptosis	Breast, lung, leukemia, prostate	Du et al., 2012; Yu et al., 2013; Liu et al., 2017; Li et al., 2018
Flavonoids	Flavonols: Quercetin, kaempferol Flavones: Apigenin, luteolin Isoflavones: Genistein, daidzein Flavanones: Naringenin, hesperetin Flavanols: Catechins (EGCG) Anthocyanins: Cyanidin, malvidin Chalcones: Phloretin, phlorizin	Onions, apple, red wine, blueberry, cherry, orange, pistachio, strawberry, citrus species	DNMT3b, HDAC1, DNMT3a DNMT1	AKT, ERK-MSK1, CD1, p53, TIMP-2, RECK, BMI1*/*c-MYC, BTG3, E2, GSTP1, WNT5a, EPHB2, RARβ, BRCA1, BRCA2 CDH1, DAPK1, MGMT, hTERT p16INK4a, hMLH1, Cip/p21, TFPI-2, ERα hTERT, Bcl-2 E6, E7	Invasion inhibition, angiogenesis decreased cell cycle arrest, cell proliferation inhibition, therapeutic cell growth inhibition, chemoprevention apoptosis	Buccal pouch tumor, colon, hepatocellular esophageal, skin, bladder, breast, prostate, cervical	Fang et al., 2003, 2005; Berletch et al., 2008; Kim et al., 2008; Majid et al., 2008, 2010; Tan et al., 2009; Li et al., 2010, 2013; Vardi et al., 2010; Wang and Chen, 2010; Adjakly et al., 2011, 2014; Kanwal et al., 2011; Meeran et al., 2011; Nandakumar et al., 2011; Priyadarsini et al., 2011; Bosviel et al., 2012; Gupta et al., 2012; Henning et al., 2012; Pandey et al., 2012; Bishayee et al., 2015; Mahmoud et al., 2015; Wang et al., 2015; Liu et al., 2016; Natarajan et al., 2016; Feng et al., 2017; Meng et al., 2017; Tseng et al., 2017; Chen et al., 2012
Stilbenes	Resveratrol	Red wine, black berry, peanuts, grape skin	DNMT, HDAC	RASSF1A, p16, APC, CCND2, AURKA, MMP9, IL8, AMY2A, MTA1, SIRT1, γ-H2AX, hTERT, BCRA-1, MDR1	Chemopreventive, apoptosis	Breast, prostate	Kai et al., 2010; Papoutsis et al., 2010; Zhu et al., 2012; Zadi Heydarabad et al., 2018; Kala et al., 2015; Medina-Aguilar et al., 2016
Lignans	Secoisolariciresinol, matairesinol, arctigenin, nordihydroguaiaretic acid (NDGA)	Tea, coffee, unrefined cereals	HDACs	Bcl2, p16INK4a	Apoptosis, cell cycle arrest	Breast	Cui et al., 2008; Hsieh et al., 2014
Tannins	Gallotannins ellagitannins	Black raspberries	ND	ND	ND	ND	
Organosulfur compounds	Sulforaphane isothiocyanates	Cauliflower, brussels sprouts, cabbage, broccoli	DNMT1 DNMT3a, DNMT3b, HDACs 1, 4, 5, and 7	Nrf2, cyclin D2, hTERT, TGFBR1, CCR4, p21^WAF1^	Chemopreventive, cell cycle arrest, apoptosis, cell growth inhibition	Prostate, breast, colon	Meeran et al., 2010; Clarke et al., 2011; Hsu et al., 2011; Rajendran et al., 2011; Zhang Y. et al., 2013; Wong et al., 2014


### Epigenetic Modulation by EGCG in Cancer Cells

Epigallocatechin-gallate is a polyphenolic catechin mainly found in green tea and its regular intake might significantly reduce the risk of breast and prostate cancer. This natural compound may induce apoptosis and inhibition of cell proliferation by epigenetic mechanisms (Greenlee, 2012; Shukla et al., 2014; Aggarwal et al., 2015). It has been reported that EGCG epigenetically reactivated p21/waf1, Bax and PUMA in prostate cancer cells, leading to cell cycle arrest and apoptosis mediated by proteasomal degradation of class I HDACs (Thakur et al., 2011). Studies of molecular modeling indicated that EGCG directly binds to the enzymatic substrates of DNMT3b and HDAC1 leading to their inhibition and reactivation of tumor suppressor genes such as retinoic acid receptor β, cadherin1 and death-associated protein kinase-1 (Khan et al., 2015).

Lee Y.H. et al. (2012) reported that EGCG repressed the hormone responsiveness of androgen receptor (AR) by reducing the acetylation of AR, leading to decreased cell proliferation and promoting cell death in LNCaP prostate cancer cell line. Remarkably, EGCG is a potential epigenetic modifier of DNMTs and HDACs and restores epigenetically silenced genes in skin and cervical cancers. For instance, in skin cancer cells, EGCG significantly decreased the proteins levels of DNMT1, DNMT3a, and DNMT3b and modulated the HDAC activities allowing the transcriptional activation of tumor suppressor genes such as p16 INK4a and Cip1/p21. In esophageal cancer, EGCG induced apoptosis and inhibited cell growth of ECa109 cells through p16 gene demethylation (Nandakumar et al., 2011; Meng et al., 2017). Moreover, EGCG reactivated the expression of WIF-1 (Wnt inhibitory factor-1) through promoter demethylation and inhibited cell growth by downregulating the Wnt canonical pathway in H460 and A549 lung cancer cell lines (Gao et al., 2009). The DNA hypomethylation as a consequence of 5-Aza-dc treatment has been shown to trigger the expression of prometastatic genes in prostate cancer. Pandey et al. (2010) showed that promoter demethylation and chromatin remodeling by green tea polyphenols leads to re-expression of GSTP1 in human prostate cancer cells.

During chemotherapy, EGCG sensitized the ERα-negative cells to respond to 17β-estradiol and the antagonist tamoxifen. EGCG in combination with the HDAC inhibitor trichostatin A (TSA) reactivated the ERα expression in triple negative MDA-MB-231 breast cancer cells by altering acetylation and methylation of histones and remodeling the chromatin structure. In addition, EGCG in combination with SFN sensitized the ERα-negative breast cancer cells to tamoxifen treatment, thus inhibiting cell proliferation (Li et al., 2010, 2017b). These examples illustrate how combination of polyphenols with current cancer treatments has a great impact in the inhibition of cancer hallmarks through epigenetic mechanisms suggesting clinical implications in patients therapies.

### Epigenetic Modulation by Curcumin in Cancer

Curcumin is a phenolic component isolated from the roots of plant *Curcuma longa* (turmeric) widely used in China and India for medicinal purposes. Several studies indicate that curcumin has antioxidant, anti-inflammatory, anti-proliferative, anti-angiogenic, and anti-cancer properties (Hewlings and Kalman, 2017; Kocaadam and Şanlier, 2017). Moreover, this natural compound has been considered as an excellent non-toxic hypomethylating agent for breast cancer therapy (Kumar et al., 2017). For instance, curcumin inhibited DNMT1 expression and restored the function of RASSF1A by promoter hypomethylation in estrogen positive MCF-7 breast cancer cell line. Furthermore, curcumin decreased the cell proliferation and breast tumors growth *in vivo* (Du et al., 2012). Curcumin and 5-aza-dc reactivated the RARβ gene through promoter hypomethylation in H460 lung cancer cells. Moreover, when A549 lung cancer cells were implanted in nude mice and treated with curcumin, tumor growth was significantly decreased. This effect was mediated by increasing of RARβ and decreasing of DNMT3b expression (Jiang et al., 2015). On the other hand, curcumin induced histone hypoacetylation and apoptosis associated to PARP activity in brain cancer cells. Also, curcumin impeded differentiation of astrocytes and promoted neural differentiation associated with hypoacetylation of H3 and H4. Other studies showed that curcumin increased protein levels of RANK in human glioblastoma cells through a demethylation mechanism. Curcumin-induced histone hypoacetylation enhanced caspase-3-dependent glioma cell death and neurogenesis of neural progenitor cells (Kang et al., 2006). Additionally, low levels of STAT3 caused RANK promoter demethylation inducing its reactivation (Wu et al., 2013).

In the acute myeloid leukemia (AML), curcumin downregulated the expression of DNMT1 in diverse cell lines *in vivo* and in *ex vivo* models. Curcumin blocks the positive regulators of DNMT1, p65 and Sp1 decreasing their activity for binding to the promoter region of DNMT1. Additionally, curcumin restored p15INK4b expression by hypomethylation of its promoter inducing cell cycle arrest at G1 phase and apoptosis *in vitro* (Yu et al., 2013). Importantly, in mice implanted with the MV4-11 cell line of AML, curcumin suppressed tumor growth (Yu et al., 2013).

In prostate cancer, curcumin inhibited tumor development in TRAMP mice model due to reversion of methylation status of Nrf2 promoter (Khor et al., 2011). Also, curcumin promoted apoptosis of LNCaP cells inhibiting JNK signaling and repressing H3K4me3 epigenetic mark. Combination of curcumin and JQ-1 efficiently suppresses prostate cancer development (Zhao et al., 2018). In HT29 colon cancer cells, curcumin inhibited the colony formation and decreased methylation of DLEC1 promoter associated to downregulation of DNMT1, DNMT3a, DNMT3b, and HDAC4/5/6/8 proteins (Guo et al., 2015a). On the other hand, Link et al. (2013) using a genome-wide approach showed that, in contrast to non-specific global hypomethylation induced by 5-aza-CdR, curcumin induced specific changes in DNA methylation of a subset of genes involved in cell viability and proliferation in colorectal cancer cells.

### Epigenetic Modulation by Quercetin in Cancer

Quercetin is a flavonoid found in fruits and vegetables such as onions, red wine, green tea, and apples. In tumor cells, quercetin blocked cell cycle and induced pro-apoptotic effects without affecting normal cells (Gibellini et al., 2011; Chirumbolo, 2013). Moreover, Xiao et al. (2011) reported that quercetin inhibited the binding of transactivators CREB2, C-Jun, C/EBPβ and NF-κB and blocked the recruitment of the coactivator p300 to COX-2 promoter. Also, quercetin inhibited p300 HAT activity, thereby attenuating the p300-mediated acetylation of NF-κB (Xiao et al., 2011). On the other hand, Tan et al. (2009) showed that quercetin inhibited tumor growth by activation of p16INK4a induced by promoter demethylation in colorectal cancer cells. In leukemic HL-60 cell line, quercetin promotes cell death by FasL expression mediated by H3 acetylation (Lee et al., 2011). Combinations of quercetin and curcumin restored protein levels of AR in androgen-receptor negative prostate cancer cells. These effects were mediated by decreasing of DNMT, resulting in global hypomethylation and induction of apoptosis via mitochondrial depolarization.

Interestingly, the synergistic effects of quercetin and curcumin combined treatment resulted in sensitization of resistant prostatic cancer cells to anti-androgen treatment (Sharma et al., 2016). In esophageal cancer, combinations of quercetin and sodium butyrate repress tumor growth and cell proliferation which was associated with downregulation of DNMT1, NF-κBp65, HDAC1, and cyclin D1. These combination inhibited HDAC through HDAC-NF-κB signaling (Zheng et al., 2014). On the other hand, quercetin exerted modulatory effects on proteins that have pivotal role in cell survival, invasiveness, angiogenesis and cell proliferation through inhibition of HDAC1 and DNMT1 in hamster buccal pouch carcinoma (Priyadarsini et al., 2011).

### Epigenetic Modulation by Resveratrol in Cancer

Resveratrol is a phytoalexin found in many plants such as blueberries, cranberries, and grapes. This polyphenol provide chemopreventive and therapeutics effects in different types of cancer regulating biological functions such as cell proliferation, cell division, apoptosis, angiogenesis and metastasis (Singh et al., 2013; Berman et al., 2017; Borriello, 2017). In DU145 (mutant p53) and LNCaP (wild type p53) prostate cancer cell lines, resveratrol downregulated the Metastasis Associated Protein 1 (MTA1) leading to destabilization of MTA1/NuRD, a nucleosome remodeling deacetylation (NuRD) corepressor complex that mediates posttranslational modifications of histones and non-histone proteins resulting in transcriptional repression (Kai et al., 2010). Downregulation of MTA1 leads to destabilization of MTA1/NuRD thus allowing acetylation/activation of p53. Additionally, combination of resveratrol and HDAC inhibitor SAHA, up-regulated p53 acetylated non-acetylated forms (Kai et al., 2010). Dhar et al. (2015) showed that resveratrol promotes acetylation and reactivation of PTEN by inhibition of the MTA1/HDAC complex, as well as inhibition of the Akt pathway. Thus, MTA1/HDAC complex is a negative regulator of PTEN, which promotes tumor cell survival and progression of prostate cancer.

In breast cancer cell lines, combination of resveratrol and pterostilbene inhibited cell proliferation, by inducing G2/M phase cell cycle arrest and apoptosis (Kala et al., 2015). The dual treatment blocked SIRT1 and decreased the expression of γ-H2AX, thus delaying the early DNA damage response. Also, these compounds cause down-regulation of DNMT1, DNMT3a and DNMT3b in HCC1806 breast cancer cells. Moreover, resveratrol and pterostilbene negatively regulated hTERT leading to inhibition of breast cancer cells growth due to the inhibition of SIRT (Kala et al., 2015). In addition, these combinations induced the reactivation of ER-α expression in ER-α negative breast cancer cells, sensitizing tumor cells to 17β-estradiol treatment which was associated to an increase in H3K9 and H4 marks in the ERα promoter (Kala and Tollefsbol, 2016).

Another study has showed the profound effects of resveratrol on global DNA methylation in breast cancer. Our group reported that resveratrol modulated the methylation status of a specific broad set of genes independently of DNMT1 inhibition in MDA-MB-231 breast cancer cells. We performed an Array-PRIMES method (aPRIMES) and DNA microarrays to screen changes in both methylome and transcriptome at genome-wide level in cancer cells treated with resveratrol at 24 and 48 h. The integrative analysis of methylome and transcriptome profiles in response to resveratrol showed that methylation alterations were concordant with changes in mRNA expression. These changes were found in several oncogenes (AURKA, CCNB1, DDIT4, DLGAP5, EYS, FAM83D, IL24, LPXN, NFIL3, PFKFB3, SLC14A1, STC1, GPR110, HK2, MMP9, NFIL3, PSMD11, RUNX2, SH3KBP1) and tumor suppressor genes (AMY2A, IL18, SLIT3, MPHOSPH9, SLC27A2, TMOD2, TTI1, and XYLB) (Medina-Aguilar et al., 2016). In human A2058 melanoma cells and MDA-MB-468 breast cancer cells, resveratrol reduced the STAT3 acetylation, inducing demethylation of the ER-α promoter region leading to its expression. Resveratrol also sensitized to antiestrogen therapy, decreasing cell viability and cell death. A similar effect was also observed in the M223 melanoma cells resistant to tamoxifen (Lee H. et al., 2012).

### Epigenetic Modulation by Sulforaphane in Cancer

Sulforaphane is a phytochemical derived from cruciferous vegetables. In cancer cells, SFN regulates cell cycle, apoptosis, tumor growth, and tumor progression (Atwell et al., 2015; Kim and Park, 2016). Gao et al. (2018) reported that the miR-9-3 promoter region is hypermethylated in lung cancer cells. Interestingly, SFN restored the miR-9-3 expression through epigenetic regulation attenuating the DNMT1 activity and DNMT3a, HDAC1, HDAC3, HDAC6, and CDH1 protein expression.

Meeran et al. (2010) reported that SFN treatment diminished cell viability and cell proliferation in breast cancer cells. Also, SFN inhibited hTERT in MCF-7 and MDA-MB-231 cancer cells, decreasing DNMT1 and DNMT3a. The downregulation of DNMTs in response to SFN induced CpG demethylation of hTERT thereby facilitating CTCF binding associated with hTERT repression. SFN increased the level of active chromatin markers H3K9ac and acetyl-H4, and suppressed H3K9me3 and H3K27me3 in hTERT promoter. Altogether, these epigenetic events induced breast cancer cells death. Moreover, combined treatments with SFN and withaferin A promoted cell death in breast cancer cells through the inhibition of DNMT1, DNMT3a, and HDAC activities in MCF-7 cells (Royston et al., 2017).

In prostate cancer cells, SFN inhibited the expression of hTERT by changes in histone acetylation and chromatin structure. In LNCaP and DU-145 cells, SFN caused an inhibitory effect in cell proliferation and induced cell cycle arrest at G0/G1 phase. In the LNCaP cell line, SFN modified the global methylation through their negative regulation of DNMT1, DNMT3a, and DNMT3b. Also, SFN induced methylation and inhibited the HDAC activities of hTERT promoter associated with H3K4me2, H3K9me3 and H3K27me3 epigenetic marks. Regarding to DU-145 cells, SFN increased the H3K18Ac and H3K4me2 marks. These modifications were linked with high risk of prostate cancer recurrence (Abbas et al., 2016). Other effects of SFN in LNCaP cells were the decrease expression of DNMT1 and DNMT3b, and the decrease methylation of cyclin D2 promoter (Hsu et al., 2011). SFN also caused demethylation of CpGs islands of Nrf2 gene promoter, which acts as regulator in the cellular oxidative stress decreasing the carcinogen-induced tumorigenesis, in TRAMP C1 cells. Moreover, SFN decreased the levels of DNMT1 and DNMT3a and downregulated the expression of HDACs 1, 4, 5, and 7, while increased the levels of the active H3ac chromatin marker (Zhang C. et al., 2013). In HeLa cervical cancer cells, SFN exerted negative effects on DNMT1 and HDAC1 activities. Ali Khan et al. (2015) showed that SNF increased the expression of RARβ, CDH1, DAPK1, and GSTP1 by restoring the CpG island methylation status and inhibition of epigenetic modulators like DNMT3b and HDAC1 in cervical cancer.

## Biological Effects of Fruit and Vegetables Consumption in Human

Fruit and vegetables and healthy lifestyles are considered protective against cancer development. Recently, The European Prospective Investigation into Cancer and Nutrition (EPIC) realized a multicenter study including 13 European countries for analyzing the association of vegetables and fruit intakes and the cancer risk development for a period from 6.5 to 8.7 years. This center concludes that consumption of vegetables and fruits do not have protector effect in patients with lung, prostate, and breast cancers. However, intake of vegetables and fruits or their combinations, or fruit juice reduced the risk of gastric, esophageal and thyroid cancer (Gonzālez et al., 2006, 2012; Boffetta et al., 2010; Linseisen et al., 2010; Zamora-Ros et al., 2018). In addition, the intake of red and processed meat were correlated with colorectal cancer and non-cardia stomach cancer risk in subjects carriers of *Helicobacter pylori*. Furthermore, the consumption of cereal fiber reduced the risk of gastric cancer mainly diffuse type (Mendez et al., 2007). In contrast, in esophageal adenocarcinoma non-significant trend was found (Gonzalez and Riboli, 2006). The effects of intake of vegetables and fruit on prevention of cancer are restricted due to the bioavailability and low concentration of these phytochemical naturals. In recent years, the use complementary and alternative medicine has increased in order to improve the quality and life expectancy of cancer patients. It is important to note that complementary medicine may be used in combination with chemotherapy, radiotherapy or other forms of therapies, but still is not considered as a standard option. However, the establishment of integrative therapies that combines standard care with alternative practices and lifestyles should be considered as a future option in preventive medicine (Molassiotis et al., 2005; Adams and Jewell, 2007; Abdallah et al., 2015).

## Conclusion and Perspectives

This review provides recent evidences of the dietary polyphenols and their effects anti-tumors by targeting the epigenetic machinery and related mechanisms. These natural phytochemical have potential benefit due to their reverting effects in the epigenetic modifications in tumor suppressor genes and oncogenes, as well as its ability to modulate and restore protein expression levels. The polyphenols cause effects in several biological functions as cell proliferation inhibition, cell cycle arrest, and induction of apoptosis in tumor cells. On the other hand, the epidemiological evidence shows that these compounds may prevents the risk of certain types of cancer development. Thus, it has been considered that these phytochemicals may increase the anti-tumor activity of standard treatments. However, the bioavailability of polyphenolic compounds in the organism is very variable depending on its chemical structure and its origin source, as well as host factors as age, gender, metabolic rate, ethnicity, etc. For all these aspects is required to establish dietary supplements in order to maintain high concentrations in plasma, for that these compounds exert its chemopreventive and therapeutic effects in cancer. Different researches show that polyphenols sensitize tumor cells through different epigenetic targets including oncogenes and tumor suppressor genes as well as DNMTs and HDACs. In addition, the combination of the polyphenols with chemotherapeutic agents exerts a synergistic effect, enhancing the effects of chemotherapeutic agents. However, it is necessary to establish more comprehensive studies that help to understand the complexity of the epigenetic pathways and their mechanisms involved in cancer, especially in highly aggressive and invasive cancers showing resistance to conventional therapies. Future research should be focused in clinical studies in humans for establishing potential personalized regimens for cancer prevention or chemotherapeutic approaches in cancer.

## Author Contributions

CL-C, ÁC-R, and JSL-G organized the entire manuscript, wrote the draft, and revised the last version of manuscript. MM-F, ÁC-R, and JSL-G wrote the introduction, epigenetic mechanisms, and phytochemicals sections. DG-R, ER-G, and HA-dlV wrote the epigenetic modulation by EGCG and curcumin in cancer sections. ÁC-R, JSL-G, and ONHdlC wrote the epigenetic modulation by quercetin, resveratrol, and SFN sections. ÁC-R, LAM and MM-F wrote the modulation of signaling pathways by polyphenols sections. Figure 1–3, as well as Table 1 were designed and made by ÁC-R, JSL-G and ONHdlC.

## Conflict of Interest Statement

The authors declare that the research was conducted in the absence of any commercial or financial relationships that could be construed as a potential conflict of interest.
